# Characterization of DNA-strand breakage induced in V79 cells by F 11782, a catalytic inhibitor of topoisomerases

**DOI:** 10.1054/bjoc.2000.1514

**Published:** 2000-12

**Authors:** J-M Barret, B T Hill, P L Olive

**Affiliations:** Division de Cancérologie, Centre de RecherchePierre Fabre, 17 avenue Jean Moulin, Castres, 81100, France; Medical Biophysics Department, British Columbia Cancer Research Centre, Vancouver, V5Z IL3, Canada

**Keywords:** F 11782, catalytic topoisomerase inhibitor, DNA-strand breaks, comet assay

## Abstract

DNA damage induced in V79 cells by F 11782, or 2″,3″-bis pentafluorophenoxyacetyl-4′,′-ethylidene-β-D glucoside of 4′-phosphate-′-demethylepipodophyllotoxin 2N-methyl glucamine salt, a novel dual catalytic inhibitor of topoisomerases I and II, was investigated using both alkaline and neutral versions of the comet assay methodology. A comparison was then made of the DNA damage induced by F 11782 with that induced by either etoposide or camptothecin under comparable experimental conditions. The results revealed that F 11782 initially induced less DNA strand breaks that either etoposide or camptothecin and rejoined such breaks more slowly. However, unlike these other drugs, the extent of DNA damage induced by F 11782 increased linearly with time of incubation. F 11782 produced both DNA single- and double-strand breaks without any clear specificity relative to phase of the cell cycle, although proliferating cells were preferentially damaged. The marked time-dependency of induction of DNA strand breaks by F 11782 may serve to explain, at least in part, its major in vivo antitumour activities. © 2000 Cancer Research Campaign http://www.bjcancer.com


*British Journal of Cancer* (2000) **83**(12), 1740–1746
© 2000 Cancer Research Campaign
doi: 10.1054/ bjoc.2000.1514, available online at http://www.idealibrary.com on IDEAL®

http://www.bjcancer.com

### Characterization of DNA-strand breakage induced in V79 cells by F 11782, a catalytic inhibitor of topoisomerases

**J-M Barret[1], BT Hill[1] and PL Olive[2]**

[1]Division de Cancérologie, Centre de RecherchePierre Fabre, 17 avenue Jean Moulin 81100 Castres, France; [2]Medical Biophysics Department, British Columbia Cancer Research Centre, Vancouver BC, Canada V5Z IL3

**Summary** DNA damage induced in V79 cells by F 11782, or 2″,3″-bis pentafluorophenoxyacetyl-4′,6′-ethylidene-β-D glucoside of 4′-phosphate-4′-demethylepipodophyllotoxin 2N-methyl glucamine salt, a novel dual catalytic inhibitor of topoisomerases I and II, was investigated using both alkaline and neutral versions of the comet assay methodology. A comparison was then made of the DNA damage induced by F 11782 with that induced by either etoposide or camptothecin under comparable experimental conditions. The results revealed that F 11782 initially induced less DNA strand breaks that either etoposide or camptothecin and rejoined such breaks more slowly. However, unlike these other drugs, the extent of DNA damage induced by F 11782 increased linearly with time of incubation. F 11782 produced both DNA single- and double-strand breaks without any clear specificity relative to phase of the cell cycle, although proliferating cells were preferentially damaged. The marked time-dependency of induction of DNA strand breaks by F 11782 may serve to explain, at least in part, its major in vivo antitumour activities. © 2000 Cancer Research Campaign http://www.bjcancer.com

**Keywords**: F 11782; catalytic topoisomerase inhibitor; DNA-strand breaks; comet assay

F 11782, or 2″,3″-bis pentafluorophenoxyacetyl-4″,6″-ethylidene-β-D-glucoside of 4′-phosphate-4′-demethylepipodophyllotoxin 2N-methyl glucamine salt (Figure 1), is a novel fluorinated lipophylic epipodophylloid (Guminski et al, 1999) with a unique mode of interaction with topoisomerases I and II (Perrin et al, 2000). In vivo, F 11782 has shown markedly superior antitumour activity against a series of experimental tumour models, compared to camptothecin or etoposide, respectively, a specific topoisomerase I- or topoisomerase II-interacting agent (Kruczynski et al, 1999). At the cellular level, F 11782 proved cytotoxic against a panel of murine leukaemia and human tumour cell lines, resulting in an accumulation of cells in the $G_2$/M phases of the cycle, with an induction of apoptosis (Etiévant et al, 1999). Preliminary studies using three different methods to detect DNA damage and various mammalian cell lines, suggested that cytotoxicity of F 11782 might result from the induction of cellular DNA damage (Barret et al, 1999; Perrin et al, 1999). Moreover, it was suggested tentatively that the DNA strand breakage induced by F 11782 might differ from that induced by etoposide. In this context, detailed characterization of DNA damage induced by F 11782 appeared important in elucidating the mode of action of this novel compound.

In this present study, the DNA damaging activity of F 11782 in cultured V79 cells has been characterized using various comet assay protocols. These results have been compared with those obtained following exposure to camptothecin or etoposide, tested concurrently, and used as reference compounds for, respectively, specific topoisomerase I- and topoisomerase II-interacting agents.

*Received 31 March 2000*
*Revised 7 August 2000*
*Accepted 13 August 2000*

*Correspondence to:* J-M Barret

### MATERIALS AND METHODS

### Chemicals and drugs

F 11782 was synthesized at the Centre de Recherche Pierre Fabre (Castres, France) and etoposide were provided by Pierre

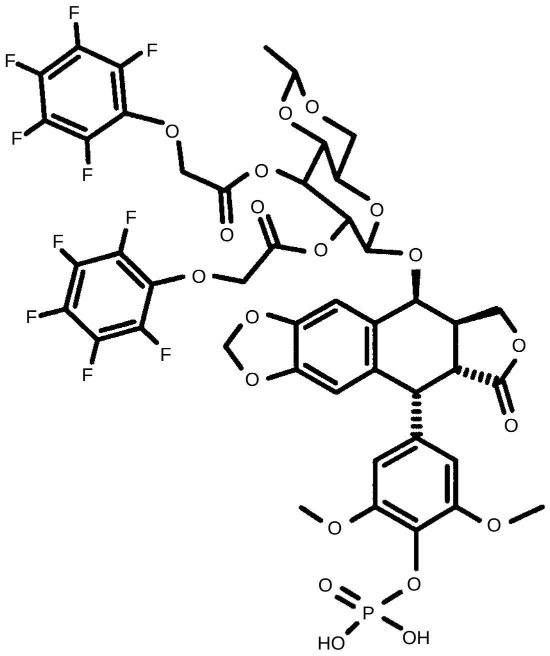

*N*-Methyl-D-glucamine salt

**Figure 1** Structure of F11782, or 2”,3”-bis pentafluorophenoxyacetyl1-4”, 6”-ethylidene-β-D-glucoside of 4´-phosphate-4´-demethylepipodophyllotoxin, 2 N-methyl glucamine

Fabre Médicament (Gaillac, France). Camptothecin was purchased from Cipla (Bombay, India). F 11782 was solubilized in water immediately prior to use, whilst etoposide and camptothecin were solubilized in dimethyl sulphoxide (DMSO) (SDS, Peypin, France) and used in assays at a maximal final concentration of 0.1%.

### Cell culture and cell treatment

Chinese hamster lung fibroblastic V79-171b cells were generously provided by Dr R Durand (British Columbia Cancer Research Center, Vancouver BC, Canada). This cell line was maintained in monolayer growth by subcultivation twice weekly in Eagle's minimal essential medium (MEM) containing 10% fetal bovine serum (FBS). For experiments, monolayer V79 cells were trypsinized for 8 minutes using 0.1% trypsin and then resuspended in 10 ml complete medium in Petri dishes at a density of $5 \times 10^5$ cells/100 mm dish. After 1 hour, cells were exposed to freshly solubilized test compounds and mixed with MEM containing 10% FBS. V79 spheroids were initiated by seeding $5 \times 10^4$ cells $ml^{-1}$ into Bellco glass spinner culture vessels containing MEM plus 5% FBS. Spheroids were fed after 3 days and daily thereafter with complete medium supplemented with antibiotics. After 14 days, spheroids (approximately 600 μm in diameter) were exposed to test compounds in suspension in MEM supplemented with 5% FBS at 37˚C using the same cell density for each experimental point. Selection of 'inner' and 'outer' cells within V79 spheroids was performed using two methods: Hoechst 33342 (Sigma, Missassauga Ont, Canada) cell sorting as described previously (Durand, 1982) or sequential trysinization (Olive et al, 1993).

### Comet assay methodologies

$2 \times 10^4$ cells, suspended in 0.5 ml ice-cold PBS, were mixed with 1.5 ml 1% low gelling temperature agarose (Sigma type VII prepared at 40˚C) in a 5 ml disposable tube. The mixture was rapidly pipetted onto a half-frosted (for the alkaline comet assay) or a fully frosted (for the neutral comet assay) microscope slide and allowed to gel for approximately 1 min at room temperature.

A cellular lysis performed under alkaline conditions led to the elimination of cell membranes and proteins and to the unwinding of the DNA from a double-stranded conformation to a single-stranded conformation. Therefore, during electrophoresis, fragments of DNA could be dissociated from the chromatid depending on whether SSBs and/or DSBs had been induced by the test drug. Thus, SSBs and DSBs were detected using an alkaline lysis solution containing 1.2 M NaCl, 30 mM NaOH, 0.5% Sarkosyl. Slides were incubated in this lysis solution for 1 hour in the dark, at room temperature. Slides were then rinsed 3 times by immersion for 20 minutes in 30 mM NaOH, 1 mM EDTA. Alkaline electrophoresis was performed in a solution of 30 mM NaOH, 1 mM EDTA at 0.6 V $cm^{-1}$ for 25 minutes. Finally, slides were neutralized with a large volume of water and stained for 20 minutes in 2.5 μg $ml^{-1}$ propidium iodide.

The use of neutral, instead of alkaline, conditions for cellular lysis led to the elimination of cell membranes and proteins, but did not allow the DNA to denature. Therefore, after neutral conditions the comet tails consist of relaxed DNA loops (Klaude et al, 1996), which migrate easier during electrophoresis when DSBs had been induced by the test drug. Thus, DSBs could be detected predominantly using the neutral comet assay. For neutral lysis, slides were carefully immersed for 4 hours in a lysing solution constituted by 30 mM EDTA, 0.5% SDS pH 8 and 0.1 mg $ml^{-1}$ of proteinase K at 50˚C. Slides were then washed 3 times in a large volume of TBE buffer (90 mM Tris, 2 mM EDTA, 90 mM boric acid pH 8.5) for 4–16 hours before performing electrophoresis at 0.6 V $cm^{-1}$ for 25 minutes. Slides were rinsed with water and stained for 1 hour in 2.5 μg $ml^{-1}$ propidium iodide.

### Image analyses

Comets were viewed using a Zeiss epifluorescence microscope combined with an intensified solid phase CCD camera and an 25 × objective, as detailed previously by Olive et al (1990). Each image of an individual comet was analysed and the following features were determined: (i) area of the comet; (ii) DNA content; (iii) percentage of DNA in the head and in the tail; (iv) mean position of the head and tail, (v) head diameter and (vi) tail length, as defined by Olive et al (1990). For each comet, all these parameters could be compared and analysed, but the two main parameters usually used are the 'tail moment' (TM), defined as the product of the percentage of DNA in the tail multiplied by the tail length and the 'DNA content', defined as the total fluorescence above background, and both these parameters were calculated. Each experiment was performed at least 3 times, and for each experimental point, 100 comets were analysed and the mean TM was calculated. Data are then presented as the mean (±SD) of these mean TM values.

### RESULTS

### Detection of DNA damage induced by F 11782, etoposide or camptothecin

Initially, the induction of DNA damage after a 1 hour exposure to either F 11782, etoposide or camptothecin, in terms of SSBs and DSBs, in V79 monolayer cells were evaluated and compared using the alkaline comet assay. F 11782 induced only limited DNA damaging activity (Figure 2). A low level of DNA-strand breaks was induced with concentrations of F 11782 up to 10 μM, but then this induction appeared to plateau or increased only slightly, irrespective of increasing concentrations of F 11782 up to 100 μM. This limited DNA damaging activity contrasted with the observed DNA damage which increased markedly with increasing concentrations from 1 to 10 μM of either etoposide or camptothecin (Figure 2). A previous study of cytotoxicity evaluated by clonogenic assay and using V79 cells showed that, after 24 hours of exposure to the drug, the cytotoxicity of F 11782 was characterized by an $IC_{50}$ value of 1.7 μM (Etiévant et al, 1999), which is consistent with the concentrations inducing DNA damage in this report. Moreover, this $IC_{50}$ value was higher than those for either etoposide or camptothecin (respectively 0.7 and 0.08 μM), evaluated concurrently, indicating that F 11782 was less cytotoxic than the two other compounds. This lower cytotoxicity may be related to the low amount of damage induced by F 11782. However, any strict relationship between cytotoxicity and the amount of damage cannot really be established since cytotoxicity depends not only on the amount, but also on the type of damage induced. Indeed a previous study by Olive and Johnston has demonstrated that the amount of strand breakage necessary to kill cells was different for each compound tested (Olive and Johnston, 1997).

© 2000 Cancer Research Campaign

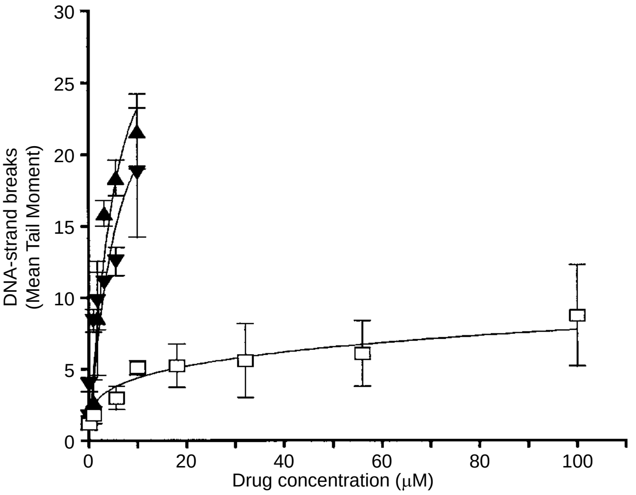

**Figure 2** Induction of DNA-strand breaks after a 1-hour drug exposure. V79 monolayer cells were exposed for 1 hour to a series of concentrations of either F 11782 (□), etoposide (▲) or camptothecine (▼) and then alkaline comet assays were performed. For each concentration of drug, mean TMs (±SD) resulting from at least three experiments containing 100 cells per experimental points were calculated

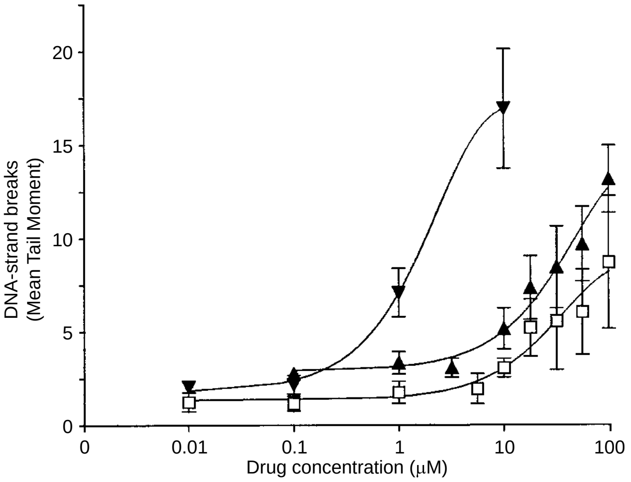

**Figure 4** Induction of DNA-strand breaks in V79 cells by F 11782. V79 monolayer cells were exposed to a series of concentrations of F 11782 for 1 (□), 4 (▲) or 12 hours (▼) and then alkaline comet assays were performed. For each experimental point, mean TMs (±SD) resulting from at least 3 experiments containing 100 cells per experimental points were calculated

### Time-dependency of DNA damage induced by F 11782

To determine whether the DNA damaging activity of F 11782 depended on time, time-course experiments were performed using V79 cells exposed to each of the three drugs and the results are shown in Figure 3. The concentration of each drug was selected so as to obtain a comparable maximum mean TM of approximately 15. Under these conditions, the number of DNA-strand breaks induced by 100 μM F 11782 increased linearly with time over 4 hours, whilst those induced by etoposide or camptothecin appeared to reach a maximum after a 1 hour incubation and then either plateaued, in the case of 3.2 μM etoposide, or tended to decrease with time, in the case of 5.6 μM camptothecin. Indeed, with camptothecin 38% of the total damage induced was no longer apparent after 4 hours of incubation. Thus, the time-dependency of DNA-strand break induction appears specific for F 11782 and is probably one factor implicated in its time-dependent cytotoxicity shown in a previous study (Etiévant et al, 1999). The rate of induction of these DNA-strand breaks induced by F 11782 was next investigated and the data in Figure 4 illustrate that this induction increased with time of exposure, up to 12 hours, using a range of concentrations of F 11782. Thus, for example, 1 μM F 11782 which did not result in any DNA-strand breakage after 1 hour, clearly induced DNA-strand breaks after 12 hours of incubation.

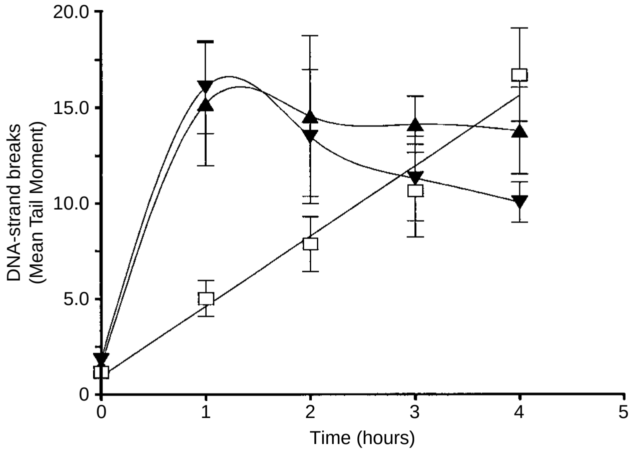

**Figure 3** Induction of DNA-strand breaks after drug exposure for 1 to 4 hours. V79 monolayer cells were exposed for various periods of time to either 100 μM etoposide (▲) or to 5.6 μM camptothecin (▼) and then alkaline comet assays were performed. These concentrations were selected so as to obtained a comparable maximum of mean TM of approximately 15. For each drug and each time of incubation, mean TMs (±SD) resulting from at least 3 experiments containing 100 cells per experimental points were calculated

### Rejoining of DNA-strand breaks induced by F 11782, etoposide or camptothecin

The time-dependent increase of DNA damage induced by F 11782 might be due to a lack of repair of such damage. To explore this hypothesis, the ability to rejoin DNA-strand breaks induced by either F 11782, etoposide or camptothecin was then investigated (Figure 5). Again, for these experiments, the concentration and the duration of exposure were selected for each drug so as to induce an amount of DNA-strand breakage corresponding to an arbitrary mean TM of 15. Following this defined exposure to the test drug, cells were then washed 3 times with fresh medium and re-incubated in drug-free medium for various times up to 1 hour. The DNA-strand breaks induced by F 11782 clearly proved reversible, decreasing linearly during the first 30 minutes of reincubation. This initial reversion though then appeared to cease after 40 minutes, with some residual DNA-strand breaks remaining detectable. With etoposide or camptothecin, the TM values fell rapidly with time before plateauing. These data therefore provide evidence of the rejoining of DNA-strand breaks induced by F 11782, etoposide or by camptothecin, under these experimental conditions. The DNA-strand breaks induced by camptothecin appeared most readily rejoined, since 70% of the damage was ‘repaired’ within 10 min, a result consistent with the fact that camptothecin induces mainly SSBs, as opposed to DSBs, and such damage is known to be repaired very rapidly (Olive et al, 1990;

*© 2000 Cancer Research Campaign*

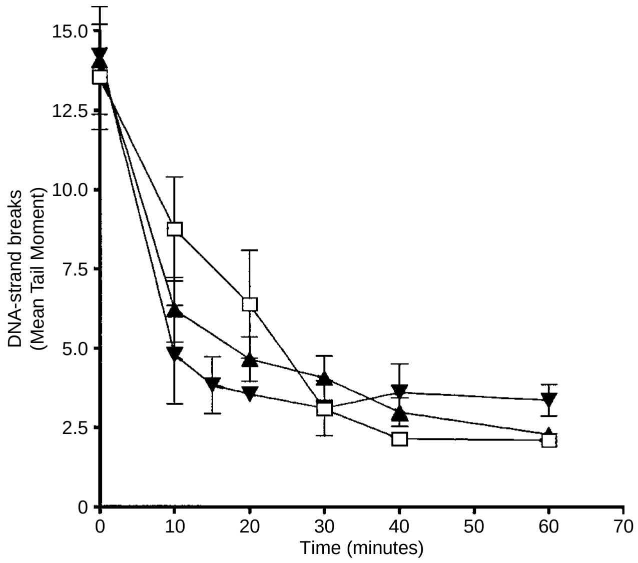

**Figure 5** Rejoining of DNA-strand breaks. V79 monolayer cells were exposed for 4 hours to 100 μM F 11782 (□) or for 1 hour to either 3.2 μM etoposide (▲) or to 5.6 μM camptothecin (▼) and then cells were washed 3 times with fresh medium and incubated at 37°C in drg-free medium for variable times before performing alkaline comet assays. These concentrations and incubation times were selected to obtain a comparable maximum of mean TM. For each incubation time and each drug, mean TMs (±SD) resulting from at least 3 experiments containing 100 cells per experimental points were calcuated

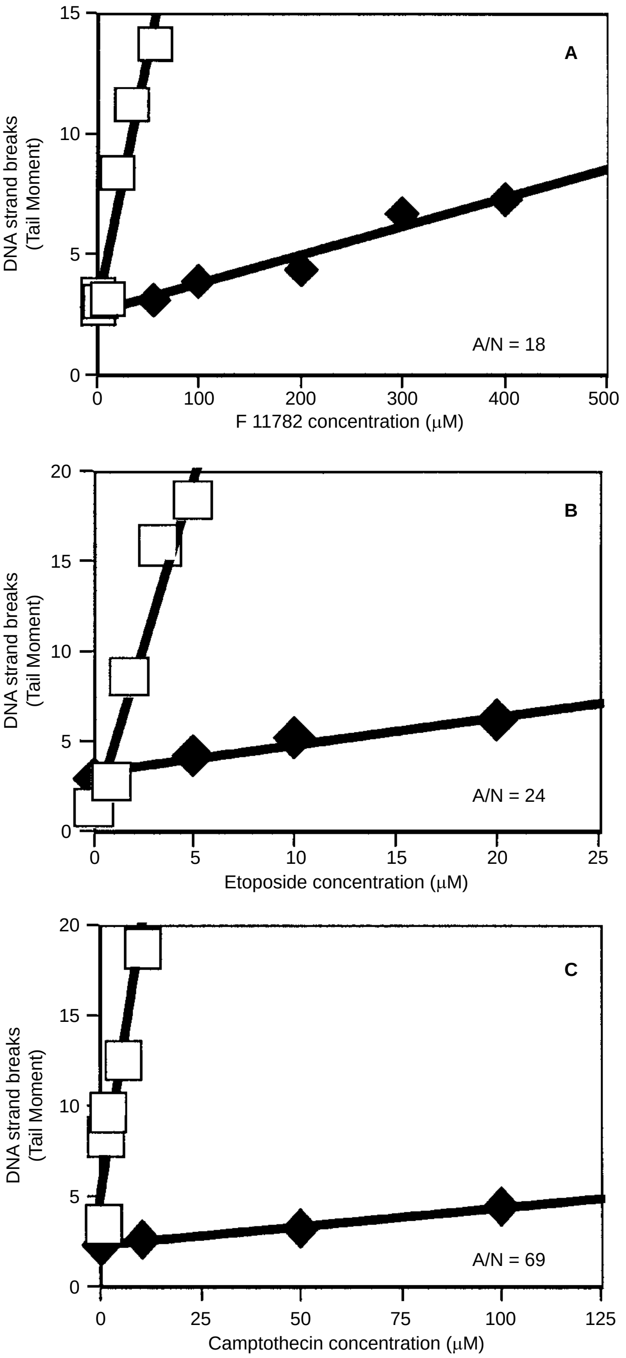

**Figure 6** Induction of SSBs and DSBs. V79 monolayer cells were exposed to various concentrations of either F 11782 (**A**), etoposide (**B**) or camptothecin (**C**) and then alkaline (□) or neutral (◆) Comet assays were performed. For each concentration of drug, mean TMs (± SD) resulting from at least 3 experiments containing 100 cells per experimental points were calculated. The SSB/DSB ratio, corresponding to the ratio of the two slopes, was indicated for each drug

Olive and Johnston, 1997). On the other hand, the DNA-strand breaks induced by F 11782 appeared to be slightly less readily rejoined, than those induced by etoposide, confirming earlier findings (Barret et al, 1999). Overall, these data suggest that the rate of strand-break repair of damage induced by the 3 drugs could be classified as following: camptothecin > etoposide > F 11782.

### Proportion of single- and double-strand breaks induced by F 11782, etoposide or camptothecin

Since SSBs and DSBs are repaired by different mechanisms which have differing kinetics, the proportion of SSBs and DSBs induced by F 11782 had next to be evaluated. Thus, the DSBs induced were evaluated using the neutral comet assay procedure, in addition to the evaluation of SSBs and DSBs using the alkaline comet assay. Under these conditions a ratio of TM for alkaline and neutral assay (A/N ratio) could be calculated. This ratio is defined by the slopes of the dose–response curves obtained using alkaline comet assays divided by those of the dose–response curves obtained using neutral comet assays. This methodology was initially described by Olive and Johnston (1997) and they defined an A/N ratio of 14 or 18 for etoposide or X-rays, both known to induce cell death mainly by their DSBs induction. With F 11782, an A/N ratio of 18 was found (Figure 6A), indicating that F 11782 induced a large proportion of DSBs. Such a result was comparable in this study with the A/N ratios of 24 obtained with etoposide (Figure 6B). Similar experiments performed using camptothecin yielded a considerably higher A/N ratio of 69 (Figure 6C), consistent with the fact that camptothecin induces predominantly SSBs.

### Cell-cycle dependency of DNA damage induced by F 11782, etoposide or camptothecin

To evaluate the hypothesis that DNA strand breaks induced by F 11782 might be accumulated though the cell cycle, the cell-cycle dependency of the DNA damaging activities of F 11782 in comparison to the two other test drugs was also investigated. Since both DNA content and DNA damage are determined for each cell,

*© 2000 Cancer Research Campaign*

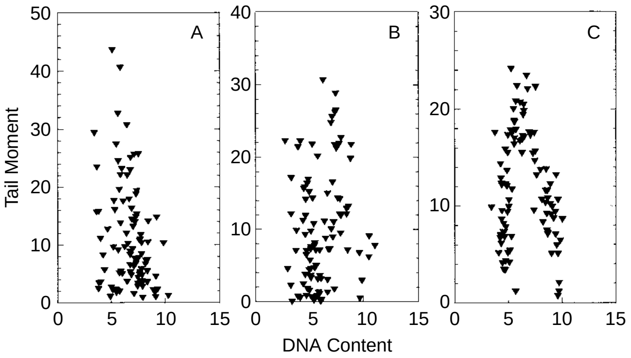

**Figure 7** Bivariate plots showing characteristic changes in DNA damage as a function of cell cycle position. V79 monolayer cells were exposed for 2 hours to 100 μM F 11782 (**A**), for 1 hour to 1.0 μM etoposide (**B**) or for 1 hour to 3.2 μM camptothecin (**C**). These conditions were selected to compare the three profiles obtained with an equivalent mean TM of 10.5 ± 1. Alkaline comet assay was performed as detailed in 'Materials and Methods' and 100 cells were analysed for each drug treatment it is possible to plot the amount of DNA damage accumulated in cells containing specific amounts of DNA, i.e. those in the different phases of the cell cycle. Figure 7 represents a comparative plot of the calculated TM versus the DNA content of a series of 100 individual cells exposed either to 100 μM F11782 (Figure 7A) for 2 hours or to 1.0 μM etoposide (Figure 7B) or 3.2 μM camptothecin (Figure 7C) for 1 hour. These experimental conditions were selected so as to obtain a comparable amount of damage by each drug, corresponding to a mean TM value of 10.5 (±1). Cells with a DNA content around 5 are judged as being in the $G_1$ phase of the cell cycle, those with a content of 10 are considered in the $G_2$/M phases, whilst those with an intermediate value approximating to 7.5 are considered as being in S phase. The data shown in Figure 7 provide no evidence of any clear cell cycle specificity for the DNA damage induced by either F 11782 (Figure 7A) or etoposide (Figure 7B) under these experimental conditions. DNA damage appears slightly higher in the $G_1$ and S phases than in the $G_2$/M phases for both these test compounds, a finding previously described with etoposide (Olive and Banáth, 1993), but this apparent difference is only marginal. On the other hand, the DNA-strand breaks induced by camptothecin appeared with a clear specificity in the S phase of the cell cycle (Figure 7C).

### Detection of DNA damage induced by F 11782 in V79 spheroids

Finally, the DNA damaging activity of F 11782 in V79 spheroids was investigated since V79 spheroids were found to be 4-fold less sensitive to F 11782 than V79 cells cultured as monolayers (Etiévant et al, 1999). In this study, two populations of cells obtained from the inner and outer regions of multicellular spheroids were obtained employing the two different protocols described in the Methods section and originally detailed by Olive et al (1993). Both techniques led to similar results, but the method using Hoechst 33342 proved more appropriate for clearly selecting the inner versus the outer populations of cells. Using this protocol, the DNA damage induced in these 'inner' or 'outer' cell populations following treatment with F 11782 or water, as the solvent control, is depicted in Figure 7. Means of TMs corresponding to the 'inner' and 'outer' cells, tested with water, were respectively 1.1 ± 0.4 and 1.7 ± 0.4, indicative of the basal levels of DNA-strand breaks in these two populations. With the 'outer' population of cells, the higher mean TM value obtained (1.7 versus 1.1 with the 'inner' cells) was consistent with the higher replicative activity of this cellular population. On the other hand, after a 4-hour incubation of spheroids with 300 μM F 11782, the mean TMs measured for the 'inner' and 'outer' populations of cells were respectively 2.2 ± 1.2 and 7.4 ± 2.4. These data therefore suggest that F 11782 induces DNA-strand breaks preferentially in the 'outer' population of cells, namely the replicative cells.

Nevertheless, the differential activity of F 11782 against 'inner' and 'outer' cells might be related to other factors which remained to be investigated like oxygenation, nutrient supply, etc. (Sutherland, 1988). However, interestingly, a difference in DNA damaging activity and cytotoxicity between the 'inner' and 'outer' cells of V79 spheroids has also been described for etoposide in earlier studies (Olive et al, 1993, 1997).

### DISCUSSION

F 11782 is a novel dual inhibitor of topoisomerase I and II which showed in vitro inhibitory activity against the DNA binding of topoisomerases without the stabilization of cleavable complexes, unlike the topoisomerase poisons (Perrin et al, 2000). Consequently, F 11782 was considered as a novel catalytic inhibitor of these topoisomerases. However, F 11782 appeared a somewhat atypical catalytic inhibitor since DNA-strand breaks were observed in cells treated by F 11782 (Barret et al, 1999). Moreover, the appearance of DNA-strand breaks was not related to protein/DNA complex formation (Perrin et al, 1999), but appeared to be correlated with the cytotoxicity of F 11782 (Barret et al, 1999). At this time, the relationship between the activities of F 11782 vis-a-vis the purified topoisomerases and the appearance of cellular DNA-strand breaks remained unclear. Thus, a characterization of DNA-strand breaks induced by F 11782 appeared important in terms of elucidating its precise mode of action.

The DNA damage induced by F 11782 in V79 cells was investigated using both alkaline and neutral versions of the comet assay. These experiments demonstrated that F 11782 initially induced only limited DNA damaging activity, as observed in a previous study with other cell lines and using different detection techniques (Barret et al, 1999; Perrin et al, 1999). However the number of DNA-strand breaks induced in V79 cells by F 11782 increased linearly with time. These results indicate that the amount of DNA-strand breakage induced by F 11782 accumulated with respect to time and was not dependent only on the concentration of F 11782. Thus, the DNA-damaging activity of F 11782 appeared to involve a cumulative cellular process, the nature of which remains to be determined. A study of the DNA damaging activity of F 11782 in V79 spheroids suggested that F 11782 induced DNA-strand breaks preferentially in the 'outer' population of cells, namely the replicative cells, implying that the DNA-strand breaks induced by F 11782 involved protein(s) involved in cell replication. Such a result would be consistent with an interaction of F 11782 with topoisomerases, without ruling out additional interaction(s). Moreover, DNA strand-breaks induced by F 11782 appeared throughout the cell cycle without any specificity for one particular phase of cycle.

Thus, this induction of DNA-strand breaks by F 11782 did not involve a cell cycle phase-dependent process. On the other hand, these DNA-strand breaks included a larger proportion of DSBs

 © 2000 Cancer Research Campaign versus SSBs, comparable to damage induced by X-rays (Olive and Johnston, 1997) and such damage appeared reversible at least in part, as shown by the rejoining of DNA-strand breaks following incubation in drug-free medium. To assess the significance of this DNA damage induced by F 11782 in terms of its dual inhibitory activity against topoisomerases I and II, a comparison was made of the DNA-strand breakage induced by F 11782 with that induced by either camptothecin or by etoposide, two topoisomerase poisons which induce predominantly and respectively SSBs and DSBs.

A comparison of the damage resulting from exposure to F 11782 with that induced by camptothecin clearly demonstrated their different nature. The appearance and reversibility of strand-breaks induced by camptothecin was more rapid than those induced by F 11782. These results relate to the nature of the DNA-strands breaks induced, since with camptothecin the proportion of SSBs induced was much greater than those induced using F 11782. Furthermore, whilst the DNA-damaging activity of camptothecin is clearly concentrated during the S phase of the cell cycle, no such cell cycle specificity was observed with F 11782. In conclusion, the mechanism(s) leading to DNA-strand breaks and to cytotoxicity are certainly different vis-a-vis F 11782 and camptothecin. Such data would be expected if F 11782 was shown to inhibit topoisomerase I by a mechanism different from that of camptothecin (Perrin et al, 2000), which might not be essential for its cytotoxicity. Complementary studies using cell lines expressing different amounts or mutated form of topoisomerase I to further elucidate this aspect are ongoing.

The nature of DNA-strand breaks induced by F 11782 and etoposide though were less easily distinguishable. Indeed, the DNA-damaging activities of these two drugs throughout the cell cycle were similar and the proportion of SSBs and DSBs induced were essentially comparable. DNA-strand break induction by F 11782 and etoposide was also similar in relation to their relative effects versus ‘inner’ and ‘outer’ cells of V79 spheroids. Nevertheless, one major difference has been highlighted between the DNA damage induced by F 11782 and that induced by etoposide and this involves the time-dependency of the appearance of DNA-strand breaks. In parallel, uptake studies using P388 cells the rate and extent of accumulation of F 11782 and etoposide were comparable (data not shown). So a slower or reduced accumulation of F 11782 is unlikely to explain this time dependency. Nevertheless, the fact that DNA damage induced by F 11782 increased with the time of incubation strongly suggested that the appearance of DNA-strand breaks resulted from a mechanism which came into play after the preliminary interaction between F 11782 and topoisomerase, whilst those induced by etoposide occurred immediately after its addition and resulted from the stabilization of cleavable complexes. Thus, unlike etoposide, the first event which leads to cytotoxicity with F 11782 might not be the induction of DNA damage. The slower rate of rejoining of the DNA-strand breaks induced by F 11782, as opposed to etoposide, is probably insufficient to explain such a differential accumulation of DNA-strand breaks with these two test compounds. Nevertheless, the reversion of DNA-strand breakage was measured in the absence of F 11782. Thus, in addition to its DNA-damaging activity, an inhibitory effect of F 11782 on DNA-strand break repair mechanisms cannot be ruled out. Such an activity has already been suggested by a previous study using DNA repair deficient cells (Barret et al, 1999), where xrs-6 cells, deficient in Ku 86 and thus deficient in non-homologous recombination, were shown to be 3-fold more sensitive to F 11782 than the parental CHO-Kl cells, whilst they were around 10-fold more sensitive to etoposide. Thus, the presence of Ku only partially influenced the cytotoxicity of F 11782 in these cells. This finding could be explained not only in terms of a different type and/or a different amount of damage being induced by these two drugs, but also by a concomitant and selective effect of F 11782 on the Ku-dependent repair pathway. Thus, F 11782 which can induce a low level of DNA-strand breaks, coupled with its interference with DNA repair process could lead to an accumulation of DNA-strand breaks with time. Such an hypothesis remains to be confirmed by testing F 11782 against cells deficient in different repair pathways and against cells deficient in other genes involved in non-homologous recombination, the predominant DBSs repair pathway. Since F 11782 has already proved a potent inhibitor of topoisomerases I and II, inhibitory activity against topoisomerase III also cannot be ruled out. Such an inhibition might explain the influence of F 11782 on DBSs repair since the activity of topoisomerase III was recently found to be related to DNA recombination (Nitiss, 1998; Ng et al, 1999). This aspect remains to be explored, but, nevertheless, the time-dependent activity of F 11782 is certainly a crucial factor in its apparently unique mechanism of action and may account, at least in part, for its high level of in vivo antitumour activity (Kruczynski et al, 1999).

### ACKNOWLEDGEMENTS

The authors wish to thank Judit Banáth for her technical assistance and Drs Chantal Etiévant, Anna Kruczynski and Dominique Perrin for their critical reading of this manuscript. This study was carried out during a period of sabbatical leave by Dr Jean-Marc Barret at the British Columbia Cancer Research Center, Vancouver BC, Canada.

### REFERENCES

Barret J-M, Montaudon D, Etiévant C, Baudouin C, Limouzy A, Cabrol N, Charvéron M, Robert J and Hill BT (1999) DNA damage induction and DNA repair inhibition by F 11782, a novel catalytic dual inhibitor of topoisomerases I and II. *Proc Am Assoc Cancer Res* **40**: 761

Durand RE (1982) Use of Hoechst 33342 for cell selection of multicell systems. *J Histochem Cytochem* **30**: 117–122

Etiévant C, Kruczynski A, Perrin D, Cabrol N, Cassabois V, Chansard N and Hill BT (1999) *In vitro* cytotoxic effects of F 11782, a novel catalytic dual inhibitor of topoisomerases I and II. *Proc Am Assoc Cancer Res* **40**: 759

Guminski Y, Cugnasse S, Fabre V, Monse B, Kruczynski A, Etiévant C, Hill BT and Imbert T (1999) Synthesis and antitumor activity of a novel epipodophylloid: F 11782, a dual inhibitor of topoisomerases I and II. *Proc Am Assoc Cancer Res* **40**: 4510

Klaude M, Eriksson S, Nygren J and Ahnström G (1996) The comet assay: mechanisms and technical considerations. *Mutat Res* **363**: 89–96

Kruczynski A, Astruc J, Chazottes E, Ricome C, Berrichon G, Imbert T, Colpaert F and Hill BT (1999) Preclinical antitumor activity of F 11782, a novel catalytic dual inhibitor of topoisomerases I and II. *Proc Am Assoc Cancer Res* **40**: 757

Ng S-W, Liu Y, Hasselblatt KT, Mok SC and Berkowitz RS (1999) A new human topoisomerase III that interacts with SGS1 protein. *Nucleic Acids Res* **27**: 993–1000

Nitiss JL (1998) Investigating the biological functions of DNA topoisomerases in eukaryotic cells. *Biochim Biophys Acta* **1400**: 63–81

Olive PL and Banáth JP (1993) Induction and rejoining of radiation-induced DNA single-strand breaks: “tail moment” as a function of position in the cell cycle. *Mutat Res* **294**: 275–283

Olive PL, Banáth JP and Durand RE (1990) Heterogeneity in radiation-induced DNA damage and repair in tumor and normal cells measured using the ‘Comet assay’. *Radiat Res* **122**: 86–94

*© 2000 Cancer Research Campaign*

Olive PL, Banáth JP and Evans HH (1993) Cell killing and DNA damage by etoposide in Chinese hamster V79 monolayers and spheroids: influence of growth kinetics, growth environment and DNA packaging. *Br J Cancer* **67**: 522–530
Olive PL, Banáth JP and Durand RE (1997) Detection of subpopulations resistant to DNA-damaging agents in spheroids and murine tumours. *Radiat Res* **375**: 157–165
Olive PL and Johnston PJ (1997) DNA damage from oxidants: influence of lesion complexity and chromatin organization. *Oncol Res* **9**: 287–294
Perrin D, Kruczynski A, Barret J-M, Etiévant C, Chansard N, Gras S, Limouzy A, Rigaud S and Hill BT (1999) Biological characterization of the *in vitro* activities of F 11782, a novel catalytic dual inhibitor of topoisomerases I and II. *Proc Am Assoc Cancer Res* **40**: 756
Perrin D, Van Hille B, Barret J-M, Kruczynski A, Etiévant C, Imbert T and Hill BT (2000) F 11782, a novel epipodophylloid non-intercalating dual catalytic inhibitor of topoisomerases I and II with an original mechanism of action. *Biochem Pharmacol* **59**: 807–819
Sutherland RM (1988) Cell and environment interactions in tumor microregions: The multicell spheroids model. *Science* **240**: 177–184

© 2000 Cancer Research Campaign
